# Genetic Characterization of *mcr-1*-Positive Multidrug-Resistant *Salmonella enterica* Serotype Typhimurium Isolated From Intestinal Infection in Children and Pork Offal in China

**DOI:** 10.3389/fmicb.2021.774797

**Published:** 2022-01-10

**Authors:** Haoran Zhang, Ying Xiang, Yong Huang, Beibei Liang, Xuebin Xu, Jing Xie, Xinying Du, Chaojie Yang, Hongbo Liu, Hongbo Liu, Hui Wang, Mingjuan Yang, Ligui Wang, Xiaofeng Hu, Lianqun Jin, Jinsong Li, Yongqiang Jiang, Shaofu Qiu, Hongbin Song

**Affiliations:** ^1^Academy of Military Medical Sciences, Beijing, China; ^2^Center for Disease Control and Prevention of PLA, Beijing, China; ^3^School of Life Sciences, Tsinghua University, Beijing, China; ^4^Shanghai Municipal Center for Disease Control and Prevention, Shanghai, China

**Keywords:** plasmid, colistin, *Salmonella* typhimurium, multidrug-resistant (MDR), bioinformatic analysis

## Abstract

With the rapid emergence of plasmid-mediated colistin resistance gene *mcr-1*, the increased resistance of *Salmonella* has attracted extensive attention. This study reports on 11 multidrug-resistant *Salmonella enterica* serovar Typhimurium strains harboring *mcr-1* in China. They all presented resistance to colistin, and additionally, one that was isolated from a child’s stool sample was also resistant to ceftriaxone and azithromycin. We screened 1454 strains of *Salmonella* for *mcr-1* gene through PCR, and these strains are all preserved in our laboratory. Antimicrobial sensitivity analysis was carried out for the screened *mcr-1* positive strains. Genetic polymorphism analysis of *S.* Typhimurium was performed by using the Pulsed-Field Gel Electrophoresis (PFGE). The plasmids harboring *mcr-1* were identified by S1-PFGE and southern blotting. Plasmid conjugation assays were used to analyze the transferability of colistin resistance. The plasmids harboring *mcr-1* were characterized by sequencing and bioinformatic analysis. Eleven *S.* Typhimurium strains harboring *mcr-1* with colistin resistance (MICs 4μg/ml) were detected, which were isolated from children and pig offal in China. All of them were multidrug-resistant strains. PFGE results revealed that the strains isolated from different samples or locations have identical genotypes. S1-PFGE and southern blotting experiments showed that three plasmids of different sizes (33, 60, and 250 kb) all carried the *mcr-1* gene. The plasmid conjugation assays revealed that *Salmonella* acquired *mcr-1* harboring plasmids by horizontal transfer. Sequencing and plasmid type analysis revealed that these plasmids were types IncX4, IncI2, and IncHI2. Among them, IncX4 and IncI2 plasmids had extremely similar backbones and contained one resistant gene *mcr-1*. IncHI2 plasmid contained multiple resistant genes including *bla*_CTX–M_, *oqxB*, *sul*, *aph*, *aadA*, and *bla*_TEM_. We identified 11 *mcr-1* harboring *S.* Typhimurium strains in China and described their characteristics. Our findings indicate that the *mcr-1* gene can effectively spread among intestinal bacteria by horizontal transfer of three types of plasmids. Moreover, the IncHI2 plasmid can also mediate the transfer of other drug resistance genes. These results reveal that constant surveillance of *mcr-1* harboring *S* Typhimurium is imperative to prevent the spread of colistin resistance.

## Introduction

The rise of multidrug-resistant (MDR, resistance to three or more classes of antimicrobials) bacteria poses a serious threat to public health (Kumar et al., 2014; Jain et al., 2020). *Salmonella* is one of the common pathogens that can cause bacterial intestinal infections and diarrhea in developed and developing countries (Lokken et al., 2016). *Salmonella enterica* serovar Typhimurium (*S.* Typhimurium), one of the most prevalent serovars of *Salmonella*, is regularly linked to human infections and is frequently reported to be associated with human infections in several industrialized countries (Gomes-Neves et al., 2012), which can result in gastroenteritis and bacteremia. For clinical therapy of *Salmonella* infection, fluoroquinolones, azithromycin, and cephalosporins have been indicated. However, the extensive use of antibacterial medicines has resulted in the emergence of *S.* Typhimurium being resistant to antibiotics (Zhu et al., 2017; Wang Y. et al., 2020).

Polymyxin, a colistin antibiotic, acts as the last-line defense against severe infections caused by broad-spectrum active gram-negative bacteria (Lu et al., 2019). Additionally, colistin resistance has developed in *S.* Typhimurium, involving a variety of mechanisms. The plasmid-mediated colistin resistance gene *mcr-1* was first discovered in *E. coli* in China in 2015 (Liu et al., 2016), and has been the subject of research attention due to the *mcr-1* gene’s ability to spread horizontally between bacteria. The colistin resistance gene *mcr-1* in the IncI2 plasmid encodes a phosphoethanolamine transferase, which is the modification of the lipid A and provides adequate protection from colistin. Multiple plasmids were used to propagate the colistin resistance gene *mcr-1*, including IncHI1, IncHI2, IncI2, IncX4, IncF, IncFI, IncFII, and IncP (Zurfluh et al., 2017; Touati and Mairi, 2021). These findings indicate that horizontal transfer of multiple resistance genes in the intestine bacteria may result in bacterial resistance. This study performed a screening analysis for the *mcr-1* gene of *S.* Typhimurium, which was preserved in the laboratory. The present study aimed to characterize the *S.* Typhimurium harboring *mcr-1* plasmids isolated from patients and food.

## Materials and Methonds

### Bacterial *mcr-1* Gene Screening, Serotyping

To clarify the epidemic situation of the colistin resistance gene *mcr-1* in critical areas in China, we detected 1454 *S.* Typhimurium strains stored in our laboratory. All *S.* Typhimurium strains were isolated from stool samples of patients and food in markets, which were collected from Shanghai City (1046), Guangdong Province (209), and Guangxi Province (199) from 2006 to 2018, respectively. These strains were strictly identified by biochemical tests (API 20E system; bioMérieux Vitek, Marcy-L’Etoile, France) and serotyped on slides by microtiter agglutination tests for O and H antigens (SSI, Copenhagen, Denmark) according to the manufacturer’s instructions. We screened all historical *S.* Typhimurium strains for *mcr-1* gene by PCR using the published primers sequence according to a study by Liu et al. (2016).

### Antimicrobial Susceptibility Testing

Antimicrobial susceptibility testing was designed using broth microdilution in Sensititre Gram Negative AST Plates for *Salmonella* strains (Thermo Fisher Scientific, Inc., West Sussex, United Kingdom) including 14 different antimicrobials: ceftriaxone (CRO), tetracycline (TE), ceftiofur (XNL), cefoxitin (FOX), gentamicin (GEN), ampicillin (AMP), chloramphenicol (CHL), ciprofloxacin (CI), trimethoprim/sulfamethoxazole (SXT), sulfisoxazole (SX), nalidixic acid (NAL), streptomycin (SM), azithromycin (AZI), and amoxicillin/clavulanic acid 2:1 ratio (AUG2). The susceptibility to polymyxin is to use the dye WST (Dojindo Molecular Technologies, Inc., Japan) by a microbial viability assay kit. A reference strain of *Escherichia coli* ATCC 25922 strain was performed in the test as quality control (Wang et al., 2017).

### Pulsed-Field Gel Electrophoresis (PFGE), S1-PFGE, and Southern Blotting

Genomic polymorphism analysis of *Salmonella* strains was performed using the pulsed field gel electrophoresis (PFGE) after a slight modification of the pulseNet standardized PFGE protocol for *Salmonella* (Ribot et al., 2006). To study the relationship between strains, *mcr-1-*negative *S.* Typhimurium Bacteria at different times in the same location and at the same time in other regions were used as the reference bacteria in the laboratory. These isolates were digested with *Xba*I (Takara, Dalian, China) at 37°C, and the *Salmonella enterica* var. Braendrup H9812 strain was used as the reference. Electrophoresis performed on a CHEF MAPPER variable angle system (Bio-Rad, California, America) with the parameters set at 2.16–63.8 s for 19 h performed following previously described methods (Liu et al., 2018). The plasmid profiles were characterized by S1-PFGE. The endonuclease S1 nuclease (Takara, Dalian, China) was used to digest at 37°C, and electrophoresis running set at 0.22–26.29 s for 15 h. The images were captured by a Gel Doc 2000 system (Bio-Rad), and imported into the BioNumerics software (v6.0) database for further processing and analysis. The southern blotting with digoxigenin-labeled *mcr-1* probe using published primer sequences (Liu et al., 2016) was performed to membrane transfer, molecular hybridization, and probe detection following a previously reported method (Zou et al., 2015).

### Plasmid Conjugation Assays

To verify the *mcr-1* positive plasmid’s transfer capacity, plasmid conjugation experiments were performed by utilizing a standard *E. coli* J53 as the recipient, and the *mcr-1* positive *S.* Typhimurium strains as donors. The donor bacteria cultured overnight were mixed with the recipient bacteria in a ratio of 1:3 and harvested, re-suspended in 80μL. The mixture was incubated for mating at 37°C for 12–18 h in 5 ml LB liquid broth. Then a Muller-Hinton agar (BD Biosciences, San Jose, CA) plate containing 100 mg/L sodium azide and 2 mg/L polymyxin B was to a selective medium for *E. coli* J53 transconjugants. Putative transconjugants were confirmed by antimicrobial susceptibility testing and detection of *mcr-1* with PCR.

### Whole Genome Sequencing and Bioinformatic Analysis

Using Next-Generation Sequencing (NGS), we sequenced plasmids of *S.* Typhimurium harboring the *mcr-1* gene. DNA was extracted from the overnight cultured strains using the QIAamp DNA Mini Kit (Qiagen, Hilden, Germany). The mate-pair library was constructed by nucleic acid protein analyzer Qsep100 to obtain DNA fragments (not less than 500 bp, not more than 800 bp) and sequenced by MiSeq sequencer. The raw reads were assembled into draft continuous sequences (contigs) by Newbler (Arredondo-Alonso et al., 2018) and NxTrim (O’Connell et al., 2015), and then spliced with Cytoscape’s GapFiller. Complete plasmid genomes were annotated using the online annotation server RAST. Identification of insertion sequence (IS), plasmid replicons, and resistance genes were performed by ISfinder,^1^ PlasmidFinder,^2^ and ResFinder (Bortolaia et al., 2020), respectively. Multiple plasmids were compared by Mauve, Brig, and CLC Genomics Workbench. The circled figure of multiple plasmids for comparison was drawn by DNAploter (Roberts et al., 2008).

## Results

### Antimicrobial Susceptibility Testing of *S*. Typhimurium Harboring *mcr-1*

Among the 1454 strains of *S.* Typhimurium maintained in our laboratory, 11 strains harboring colistin resistance gene *mcr-1* were identified. Eight strains were isolated from the feces of children under the age of five, while the remaining strains were isolated from pork offal. These strains exhibited multidrug resistance, including polymyxin (MICs 4μg/ml). Additionally, the majority (63.6%) of *S.* Typhimurium harboring *mcr-1* were resistant to the third-generation cephalosporins. Notably, one of them exhibited co-resistant to azithromycin and third-generation cephalosporins (Table 1).

**TABLE 1:** Characteristic of 11 *mcr-1-*positive MDR *S.* Typhimurium.

Strain no.	Antibiogram^a^	Results of sequencing for 11 *mcr-1-*positive plasmids				
		Plasmid name	Size of *mcr-1* plasmid (b)	Type of *mcr-1* plasmid	Drug-resistant gene	IS types
S49	CRO,EFT,AMP,GEN, SM,SX,CHL,COL	pS49	222,291	IncHI2	*mcr-1*, *bla*_CTX–M_, *aac*, *floR*, *aph*, *fosA*	ISApll
S51	CRO,EFT,AMP,GEN,SM, SX,SXT,CHL,TE,COL	pS51	249,475	IncHI2	*mcr-1, bla_CTX–M_, sul*, *oqxA*, *oqxR*, *dfrA*, *floR*, *oqxB*, *aadA*, *aph*, *aac, fosA*, *cml*	ISApll
S52	CRO,EFT,AMP,GEN, SM,SX,CHL,TE,COL	pS52	249,043	IncHI2	*mcr-1*, *bla_CTX–M_*, *oqxB, sul*, *aph*, *aadA*, *fosA*, *floR*, *aac*, *cml*, *oqxR*	ISApll
S53	CRO,EFT,AMP,GEN, SM,SX,CHL,COL	pS53	228,926	IncHI2	*mcr-1*, *bla_CTX–M_*, *oqxA*, *sul*, *oqxR*, *aadA*	ISApll
S54	CRO,EFT,AMP,GEN, SM,SX,CHL,COL	pS54	222,880	IncHI2	*mcr-1*, *bla_CTX–M_*, *aac*, *sul*, *aph*, *floR*, *fosA*	ISApll
S55	FOX,AUG2,CRO,EFT, AMP,SM,SX,SXT, AZ,CHL,TE,COL	pS55	59,233	IncI2	*mcr-1*	None
S56	COL	pS56	60,454	IncI2	*mcr-1*	None
S60	AMP,NAL,GEN,SM, SX,SXT,CHL,TE,COL	pS60	33,308	IncX4	*mcr-1*	IS26
S67	AMP,NAL,SM, SX,TE,COL	pS67	33,308	IncX4	*mcr-1*	IS26
S69	AMP,NAL,GEN,SX, SXT,CHL,TE,COL	pS69	33308	IncX4	*mcr-1*	IS26
S70	CRO,EFT,AMP,GEN, SM,SX,CHL,TE,COL	pS70	223,256	IncHI2	*mcr-1*, *bla*_CTX–M_, *aac*, *sul*, *aph*, *floR*, *fosA*	ISApll

*^a^CRO, ceftriaxone; TE, tetracycline; XNL, ceftiofur; FOX, cefoxitin; GEN, gentamicin; AMP, ampicillin; CHL, chloramphenicol; CI, ciprofloxacin; SXT, trimethoprim/sulfamethoxazole; SX, sulfisoxazole; NAL, nalidixic acid; SM, streptomycin; AZI, azithromycin; AUG2, amoxicillin/clavulanic acid 2:1 ratio.*

### Pulsed Field Gel Electrophoresis, Plasmid Profiling, and Southern Blotting

We studied the PFGE results of 11 *mcr-1-*positive *S.* Typhimurium strains and 12 *mcr-1-*negative *S.* Typhimurium strains. The majority of these (10/11) belong to ST34, while one (S55) belongs to ST19. In total, 13 distinct PFGE genotypes were discovered among 23 strains using the 85% cutoff (Supplementary Figure 1). The *mcr-1* positive strains were distributed in 10 different genotypes. Furthermore, numerous isolates obtained from diverse samples and provinces were identified as belonging to the same genotype. For instance, *S.* Typhimurium harboring *mcr-1* strains isolated from patient samples and food in Shanghai and the strains isolated from patients in Zhejiang and Henan were in the same cluster. S1-PFGE analysis showed that two of 11 *mcr-1* positive *S.* Typhimurium carried two plasmids, while the remaining eight carried single plasmid (Supplementary Figure 2). Southern blotting revealed that all of them carried one plasmid harboring *mcr-1* (Supplementary Figure 2).

### Plasmid Conjugation Assays

To determine the transferability of the plasmid harboring *mcr-1*, plasmid conjugation assays were performed using 11 *mcr-1* positive *S.* Typhimurium as donors and *E. coli* J53 as the recipient. Five of eleven recipients tested positive for the resistance gene *mcr-1* via PCR amplification and sequencing analysis. Five plasmids harboring *mcr-1* were identified (pS49, pS51, pS52, pS55, pS56). Three were IncHI2 and two were IncI2 plasmids (Supplementary Figure 1). The MIC of colistin for the transconjugants (the *E. coli* J53 harboring *mcr-1* gene) was increased to 4 μg/ml, which was significantly more than the colistin resistance levels of the original J53 strains (which have MIC values of 0.125 μg/ml). It can be speculated that the transconjugants acquire the donor strains’ colistin resistance gene.

### The Complete Sequence of Plasmid Harboring *mcr-1*

**FIGURE 1:**
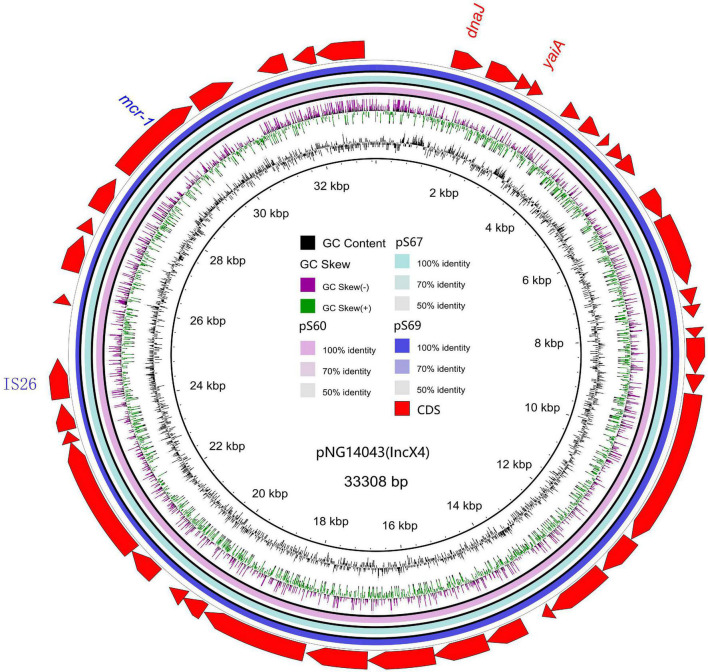
Sequencing alignment of *mcr-1* harboring IncX4 plasmids. The *mcr-1* harboring plasmid pNG14043 with GenBank no. KY120364 which was isolated from *S.* Typhimurium in Taiwan was used as refrence plasmid (black circle). The outmost circle in red arrows denots the annotations of refrence plasmid. The figure shows the extremly high degree of homology of the four *mcr-1* harboring IncX4 plasmids. Detailed information of *mcr-1* location of plasmids is provided in Figure 2.

**FIGURE 2:**
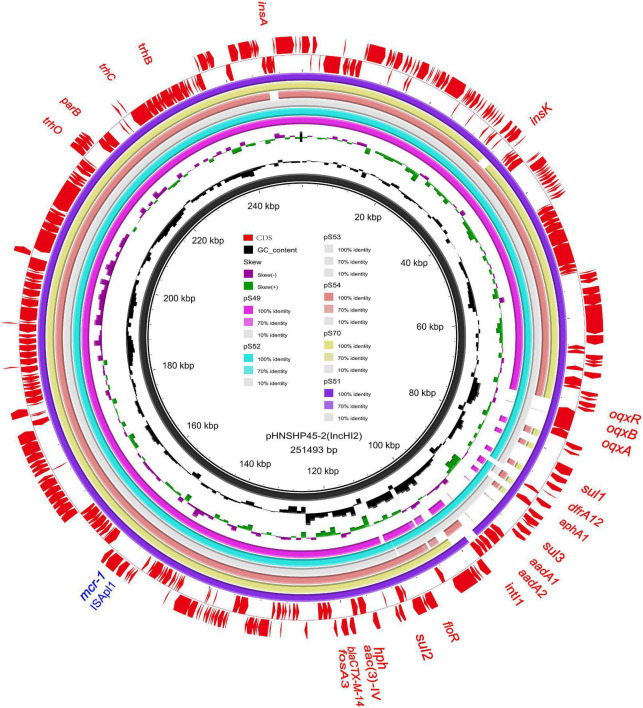
Genetic context of 11 plasmids surrounding the *mcr-1* gene. In CDS, red and blue arrows represent *mcr-1* and IS, respectively, black arrows present plasmid bone. The light blue and orange shaded regions indicate genetic regions that show the direct and reverse nucleotide identity homology between different segments (> 99%).

**FIGURE 3:**
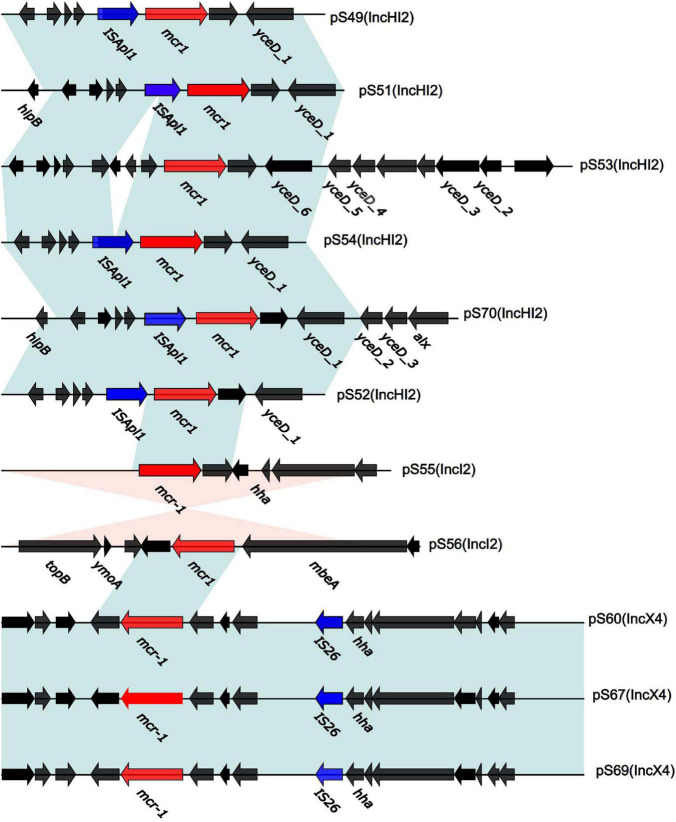
Sequencing alignment of *mcr-1* harboring IncI2 plasmids. The first *mcr-1* harboring plasmid, pHNSHP45 with GenBank no. KP347127 which was isolated from E. coli strains from Shanghai in July, 2013 was used as refrence plasmid (black circle). The outmost circle in red arrows denots the annotations of refrence plasmid. The IS683 and ISApl1 are absent in two IncI2 plasmids in this study. Detailed information of *mcr-1* location of plasmids is provided in Figure 2.

**FIGURE 4:**
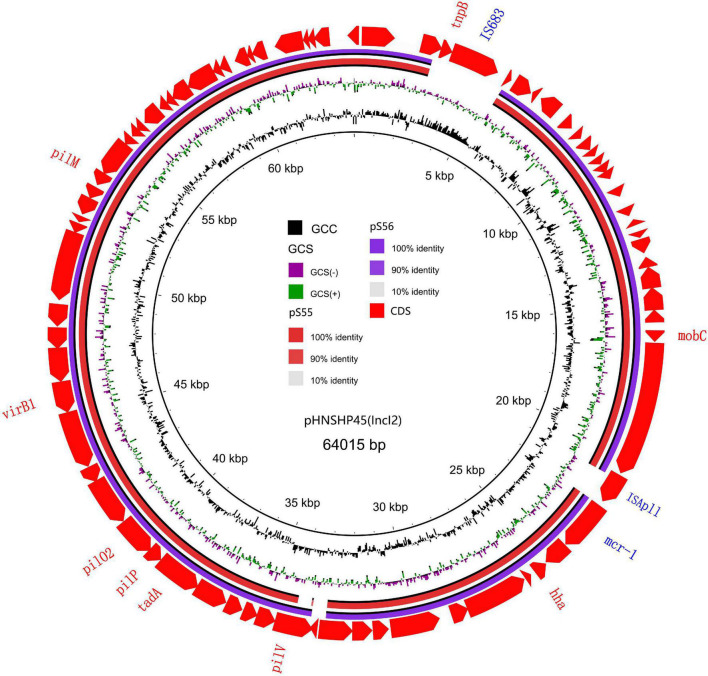
Sequencing alignment of *mcr-1* harboring IncHI2 plasmids. pHNSHP45-2 with GenBank no. KU341381 which was isolated from E. coli strains was used as refrence plasmid (black circle). The outmost circle in red arrows denots the annotations of refrence plasmid. Among the six IncHI2 plasmids, pSH51, pS52, and pS53 exhibit sequence with the refrence sequence, others are low sequence homology. An MDR region is exhibieted in the five IncHI2 plasmids. Detailed information of *mcr-1* location of plasmids is provided in Figure 2.

We sequenced the plasmid of 11 *S.* Typhimurium strains. As a control, we downloaded five highly comparable plasmids harboring *mcr-1* from *Salmonella* strains for comparison (at above 95% coverage and above 99% identity) from NCBI (full name and cited reference). Three IncX4, two IncI2, and six IncHI2 plasmids harboring the *mcr-1* gene were identified by analyzing the plasmid sequences. Plasmid sequence comparison revealed that the lengths of the three IncX4 plasmids were approximately 33 kb and the sequences were completely identical; the lengths of the two IncI2 plasmids were 59,233 and 60,454 b, respectively, and the sequence differences were within 1.3 kb, and the 6 IncHI2 plasmid sequences were around 220-250 kb in length and the length variance was less than 2.8 kb. The three IncX4 plasmids with the identical sequence had a typical IncX4 backbone and were extremely similar to pNG14043 from *Salmonella* in Taiwan (at above 99% homology). The IS26 was upstream of resistance gene *mcr-1* in our isolates. The IncX4 plasmids had only the resistance gene *mcr-1* and no other identifiable resistance genes (Figures 1, 2). In contrast to pHNSHP45, three IncX4 plasmids lacked an ISApl1 insertion element upstream of *mcr-1* but had an IS26 insertion element. The IncI2 plasmids were similar to pHNSHP45 by *E. coli* strains from Shanghai in July 2013. The sequence of *mcr-1* on these plasmids was identical to pHNSHP45. However, two IncI2 plasmids lacked an ISApl1 insertion element upstream of *mcr-1*, and an IS683 region was found to be missing in all two IncI2-type plasmids isolated in this study (Figures 2, 3). Unlike the IncX4 and IncI2 plasmids, IncHI2 plasmids exhibited the most genetic diversity (Figures 2, 4). Compared with the pHNSHP45-2 plasmid, these IncHI2 plasmids with the common backbone were 250 kb in length. All the IncHI2 plasmids contained a single copy of *mcr-1*, and the sequence surrounding *mcr-1* shared 100% sequence identity. However, these plasmids contain numerous variable resistance genes, integrons, and ISs. The reference plasmid contained a variety of resistance genes, including *bla*_CTX–M_, *oqxA*, *oqxB*, *oqxR*, *sul*, *aph*, *aadA*, *dfrA*, *floR*, *aac*, *fosA*, *hph*. The IncHI2 plasmids we investigated had different resistance genes and insert sequences with the reference plasmid (Table 1). Compared to the reference plasmid, several drug-resistant genes were missing, including *oqxA, oqxB, oqxR, sul*, *aadA* in three IncHI2 plasmids (pS49, pS54, pS70) and *dfrA12* in the five IncHI2 plasmids (pS49, pS52, pS53, pS54, pS70) (Figure 4). Five of six IncHI2 plasmids contained the insertion sequence ISApl1 on the upstream of *mcr-1*, but another plasmid lacked ISApl1 around *mcr-1* (Figure 2).

## Discussion

*Salmonella* is a widespread zoonotic pathogen that can cause human food poisoning and diarrhea (Ling et al., 2020). In general, food poisoning and diarrhea caused by non-typhoidal *Salmonella* (NTS) are self-limited. However, if the patient is young children, older people, and people with weak immune systems, antibiotic therapy will be preferred with multi-drug resistant *Salmonella* infection if the patient is a young child, elderly, or has a weak immune system. Studies have demonstrated that the multidrug resistance rate of *Salmonella* increased to 40% in the last decade of the twentieth century (Elbediwi et al., 2020). Resistance to fluoroquinolones, azithromycin, and third-generation cephalosporins in NTS species has been reported from numerous countries in the world (Tack et al., 2020; Appiah et al., 2021). Among a large number of *Salmonella* serotypes, *S.* Typhimurium and the rapid growth of multidrug-resistant has been a subject of concern globally (Huang et al., 2020). Colistin is considered as a last-line therapy for multidrug-resistant *S.* Typhimurium infection based on its prevalence and has been listed as a significant antibiotic by WHO since 2015 (Li et al., 2017). As colistin is widely used, bacteria have developed resistance to colistin. 37 *Salmonella* strains were identified harboring the *mcr-1* gene among 12,053 *Salmonella* strains collected from diarrhea outpatients under surveillance (Lu et al., 2019), and our finding appeared consistent with previous studies in Shanghai. We identified 11 *mcr-1-*positive strains among 1454 strains of *S.* Typhimurium (0.76%). Notably, eight strains of *S.* Typhimurium harboring *mcr-1* were isolated from the feces of children under the age of five (Supplementary Table 1). This observation is consistent with the finding of Luo et al. (2020), in which the majority of the *Salmonella* infection occurs in children under the age of five and patients with inadequate immunity. All *mcr-1* harboring *S.* Typhimurium strains from various sources were resistant to multiple antibiotics. In total, 63.6% of them were resistant to colistin and third-generation cephalosporin. Moreover, one of these strains was isolated from children under 5 years old was resistant to colistin, azithromycin, and third-generation cephalosporins. Considering the important role of azithromycin and third-generation cephalosporins in clinical treatment, this causes concern. ST19 and ST34 were common genotypes in *S*. Typhimurium (Zhang et al., 2021). The MDR ST34 *S*. Typhimurium has become a threat to public health due to its carriage of *mcr-1* and *mcr-3* (Biswas et al., 2019), and has been frequently detected in human clinical samples and food samples in China (Sun et al., 2014). The PFGE results indicated that *S.* Typhimurium that were isolated from various samples and provinces had identical genotypes. For example, *S.* Typhimurium harboring *mcr-1* strains isolated from children and food in Shanghai were clustered with the *S.* Typhimurium in Zhejiang and Henan strains. This result indicated that *S.* Typhimurium was prevalent in a number of regions in China. Thus, the monitoring of multidrug-resistant *S.* Typhimurium strains should significantly prevent their spread.

Plasmids play a vital role in the acquisition of colistin resistance caused by drug resistant genes (McGann et al., 2016). In 2016, China reported the first case of Plasmid-mediated colistin resistance in the form of *mcr-1* (Liu et al., 2016). Colistin resistant gene *mcr-1* was widely spread in animals, the environment, and food in a number of nations and areas throughout the world by plasmid horizontal transfer (Zurfluh et al., 2017; Huang et al., 2020; Wang Z. et al., 2020; Touati and Mairi, 2021). During plasmid transfer, the plasmid harboring *mcr*-1 exhibited significant diversity in terms of antibiotic resistance patterns, incompatibility groups, and genetic content (Touati and Mairi, 2021). In our study, three types of plasmids (IncI2, IncX4, and IncHI2) harboring *mcr-1* were identified from 11 S. Typhimurium strains. The first reported *mcr-1* gene was identified in an IncI2 Plasmid. IncI2 and IncX4 plasmids, which promote *Salmonella* resistance, are the two major types of plasmids spreading globally (Arredondo-Alonso et al., 2018). IncHI2 plasmids are well-known for their role in clinically significant antibiotic resistant genes (Hammad et al., 2019). According to a previous study on *Enterobacteriaceae* (Zingali et al., 2020), IncHI2 plasmid (216–280 kb) is the fifth most common plasmid family containing a multidrug resistance region. it is also one of the major plasmid groups harboring *mcr-1* gene variants. The present study observed a coexistence of plasmids harboring *mcr-1* and multiple drug resistance genes, including *oqxB*, *bla*_TEM_, and *bla*_CTX_ resistance genes. These genes in drug-resistant plasmids were one of the significant factors for the decreased sensitivity of colistin, quinolones, and third-generation cephalosporins. The rapid spread of antibiotic resistance in a particular area was caused by IncHI2 plasmids transfer carrying multiple drug resistance genes between bacteria. MDR IncHI2 plasmids containing *mcr-1* are widely distributed in human pathogens and are the efficient vector for the transmission of *mcr-1* and other drug-resistant genes (Hammad et al., 2019). Whether MDR IncHI2 plasmids can alter the *Salmonella* resistance phenotype and disseminate rapidly is concerning.

The insertion sequence (IS) family in the bacterial genome with a widely variable DNA sequence in nature is also one of the significant modes for resistance gene transmission between bacterial pathogens. The 11 plasmids are classified into distinct patterns based on the existence of IS elements and the connection with *mcr-1* sites. In our study, IS26 elements are closely related to the IncX4 type plasmid, while the ISApl1 element is closely related to the IncHI2 type plasmid. ISApl1 is consistently associated with the *mcr-1* gene and the *mcr-1* gene cassette can be inserted into a variety of genetic loci in different plasmids. Five of the six IncHI2 plasmids contained the ISApl1 insertion sequence located upstream of *mcr-1*, and only one plasmid lacked ISApl1 around *mcr-1*. The IncHI2 backbone structure is considered to be stable (Garcia-Fernandez and Carattoli, 2010), and the presence of different resistance genes in these plasmids is probably due to the acquisition of different mobile genetic elements (Cain and Hall, 2012). As such, the existence of ISApl1 in our study offers a potential hotspot for involving novel antibiotic resistant genes.

## Conclusion

The present study describes the genetic characterization of *mcr-1*-positive multidrug-resistant *S.* Typhimurium isolated from intestinal infection in children and pork offal in China. Our results indicated that *mcr-1*-positive *S.* Typhimurium strains were multidrug-resistant, and one strain was additionally resistant to ceftriaxone and azithromycin. Three types of plasmids harboring *mcr-1* have respective characteristics regarding IS and resistance genes. Plasmids harboring *mcr-1* and other resistance genes confer resistance to colistin and other multiple antibiotics. Therefore, the findings of this study are critical to estimating the transmission of *mcr-1* and monitoring the international epidemic.

## Data Availability Statement

The datasets presented in this study can be found in online repositories. The names of the repository/repositories and accession number(s) can be found in the article/Supplementary Material.

## Author Contributions

JL, SQ, and HS designed the study. HZ wrote the main manuscript. YX, BL, and XX participated in the specimen collection and revised the manuscript. YH and LW contributed to the bioinformatics data analysis. JX, XD, XH, and LJ participated in data collection. XD, CY, HL (9th author), HL (10th author), HW, and MY performed the experiments. YJ critically revised important knowledge content. YJ, SQ, and HS gave final approval of the version to be submitted. All authors made substantial contributions to preparation and submission of the manuscript.

## Conflict of Interest

The authors declare that the research was conducted in the absence of any commercial or financial relationships that could be construed as a potential conflict of interest.

## Publisher’s Note

All claims expressed in this article are solely those of the authors and do not necessarily represent those of their affiliated organizations, or those of the publisher, the editors and the reviewers. Any product that may be evaluated in this article, or claim that may be made by its manufacturer, is not guaranteed or endorsed by the publisher.

## Footnotes

1
https://www-is.biotoul.fr/search.php

2
https://cge.cbs.dtu.dk/services/PlasmidFinder/
