# Supplementary figures and images for: Genetic Characterization of mcr-1-Positive Multidrug-Resistant Salmonella enterica Serotype Typhimurium Isolated From Intestinal Infection in Children and Pork Offal in China
Source: Front Microbiol. 2022 Jan 10;12:774797. doi: 10.3389/fmicb.2021.774797 (PMC8784875; doi:10.3389/fmicb.2021.774797)

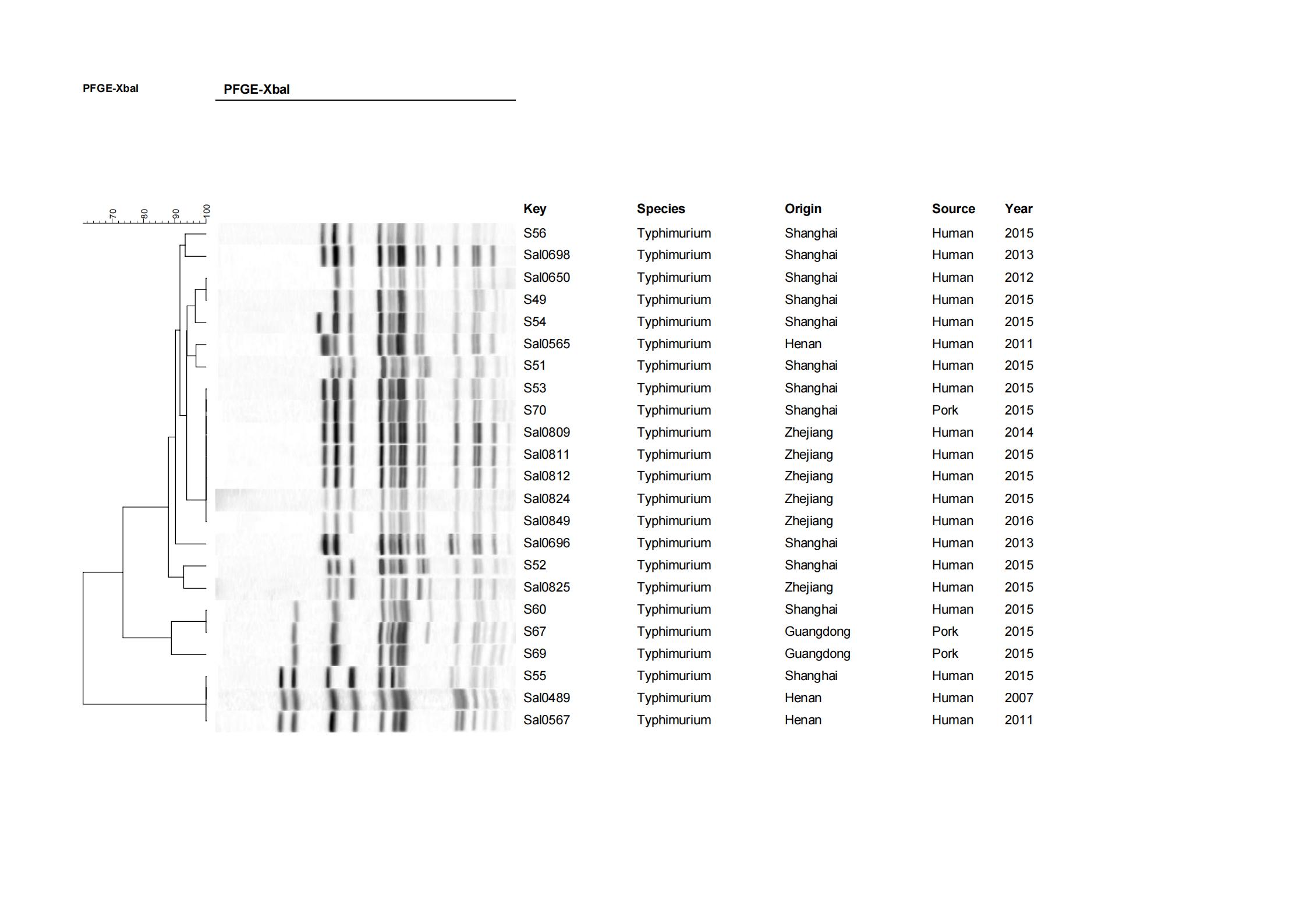

Supplement: Supplementary file 1 [file Image_1.jpg]

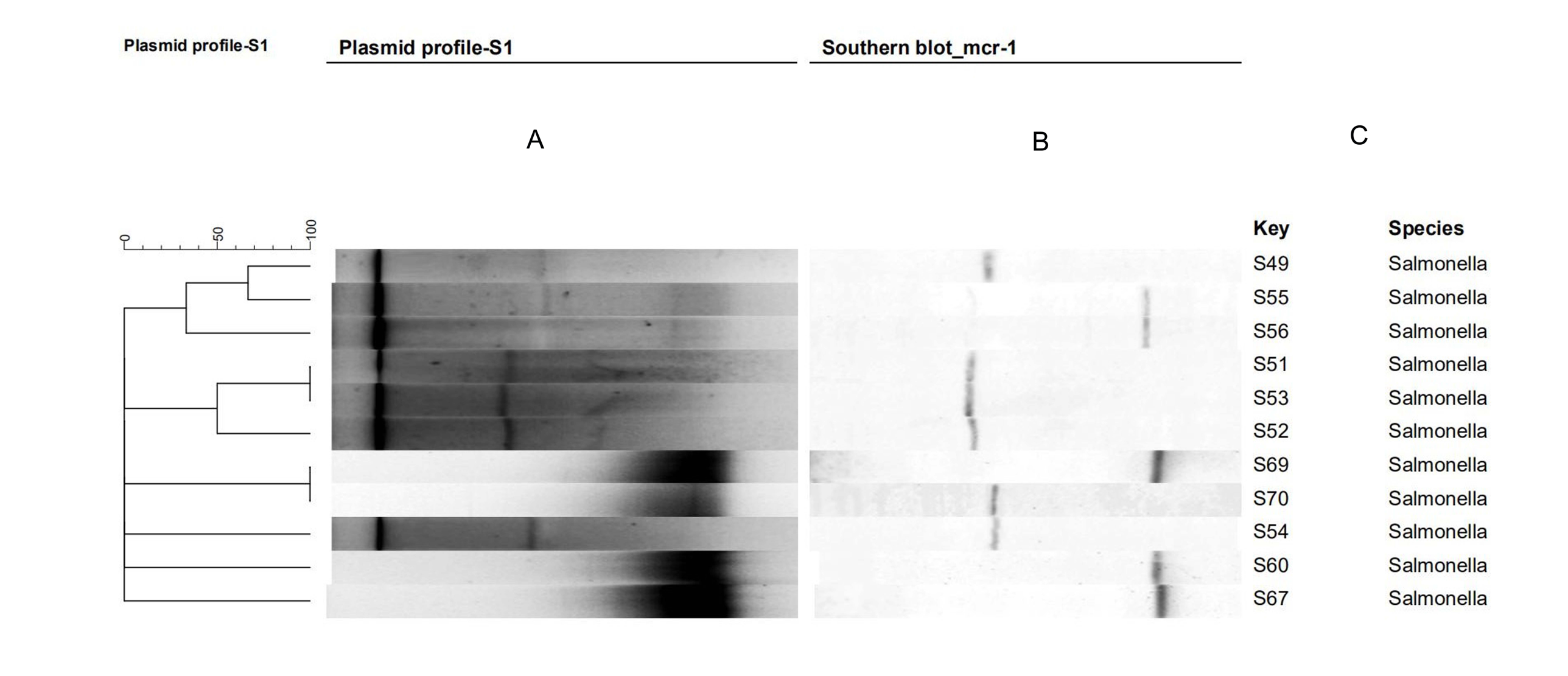

Supplement: Supplementary file 2 [file Image_2.jpg]
