# Supplementary material for: Genetic Characterization of mcr-1-Positive Multidrug-Resistant Salmonella enterica Serotype Typhimurium Isolated From Intestinal Infection in Children and Pork Offal in China
Source: Front Microbiol. 2022 Jan 10;12:774797. doi: 10.3389/fmicb.2021.774797 (PMC8784875; doi:10.3389/fmicb.2021.774797)
Supplement: Supplementary file 3 [file Table_1.doc]

***Table*** *S1 Information on source of eleven mcr-1-positive MDR S. Typhimurium samples*

| Strain no. | Collection time | Sample | Sex | Age |
| --- | --- | --- | --- | --- |
| S49 | 2015/9/7 | Human fecal | male | 1 |
| S51 | 2015/8/26 | Human fecal | female | 1 |
| S52 | 2015/9/5 | Human fecal | female | 1 |
| S53 | 2015/9/7 | Human fecal | female | 3 |
| S54 | 2015/9/7 | Human fecal | male | 1 |
| S55 | 2015/9/28 | Human fecal | male | 4 |
| S56 | 2015/10/19 | Human fecal | male | 10m |
| S60 | 2015/7/15 | Human fecal | female | 9m |
| S67 | 2015/7/28 | Pork products | none | none |
| S69 | 2015/9/12 | Pork products | none | none |
| S70 | 2015/1/20 | Pork products | none | none |

**Fig S1.** Pulsed field gel electrophoresis (PFGE) of the fifteen *Salmonella* isolates in 2015. (A) Results of PFGE profiles of *Salmonella* were clustered. (B) The key, species, origin, source and isolation year of the fifteen *Salmonella* are shown.

**Fig S2.** S1-PFGE and southern blotting of the eleven *Salmonella* isolates in 2015. (A) Results of S1-PFGE profiles of *Salmonella* were clustered. (B) Results of southern blotting profiles of *Salmonella* were clustered. (C) The key, species of the eleven *Salmonella* are shown.
